# Atezolizumab-associated encephalitis in metastatic lung adenocarcinoma: a case report

**DOI:** 10.1186/s13256-020-02411-y

**Published:** 2020-07-04

**Authors:** Yoshitaka Yamaguchi, Hikaru Nagasawa, Yuji Katagiri, Manabu Wada

**Affiliations:** 1 Department of Neurology, Yamagata Prefectural Central Hospital, 1800 Aoyagi, Yamagata, 990-2292 Japan; 2 Department of Respiratory Medicine, Yamagata Prefectural Central Hospital, 1800 Aoyagi, Yamagata, 990-2292 Japan

**Keywords:** Atezolizumab, Encephalitis, Immune checkpoint inhibitor, Immune-related adverse event, Lung adenocarcinoma, Programmed death ligand 1 inhibitor

## Abstract

**Background:**

In recent years, immune checkpoint inhibitors have been widely used as a crucial therapy in malignant tumors. Immune checkpoint inhibitors can cause various autoimmune side effects called immune-related adverse events because they generate an exaggerated inflammatory response. Encephalitis associated with atezolizumab has rarely been reported as an immune-related adverse event. A case of encephalitis caused by treatment with atezolizumab is presented.

**Case presentation:**

A 56-year-old Japanese man with lung cancer previously treated with surgery and chemotherapy was admitted with high fever, consciousness disorder, and motor aphasia. His first atezolizumab treatment was 17 days earlier. Admission brain magnetic resonance imaging with gadolinium enhancement showed no abnormalities. Cerebrospinal fluid showed cell count 20/l, protein 166 mg/dl, glucose 73 mg/dl, and interleukin 6 82.9 pg/ml (normal< 8.7 pg/ml). Atezolizumab-induced encephalitis was diagnosed. His symptoms improved the day after steroid pulse therapy was started. Following steroid pulse therapy, oral prednisolone 30 mg was started and tapered. The cerebrospinal fluid findings normalized on day 14. He was discharged on day 16 without neurological sequelae.

**Conclusion:**

In this case of encephalitis associated with atezolizumab, prompt steroid pulse therapy led to a successful response, and the outcome was good. The cerebrospinal fluid level of interleukin 6 reflected the severity of the encephalitis well. Clinicians should be aware of the possibility of encephalitis after initiation of immune checkpoint inhibitors.

## Introduction

In recent years, immune checkpoint inhibitors have been widely used as a crucial therapy for patients with malignant tumors. Malignant cells prevent attacks from activated T cell-mediated immunity by inhibitory signals from programmed death ligand (PD-L) 1 and 2, which interact with programmed death (PD) 1 expressed on activated T cells. Immunotherapies targeting these ligands have shown efficacy and safety in the treatment of advanced malignant disease. Atezolizumab, an immune checkpoint inhibitor that targets PD-L 1 and 2 is approved for the treatment of urothelial carcinoma and non-small cell lung cancer and is currently under study for the treatment of gynecological, breast, lymphoma, melanoma, urological, and colorectal malignancies [1].

Immune checkpoint inhibitors can induce various autoimmune side effects called immune-related adverse events (irAEs) because they generate an exaggerated inflammatory response [2]. Neurological irAEs associated with immune checkpoint inhibitors include myasthenia gravis, Guillain-Barré syndrome, peripheral neuropathy, autonomic neuropathy, aseptic meningitis, encephalitis, and transverse myelitis [2]. However, encephalitis associated with atezolizumab has rarely been reported as an irAE. A case of encephalitis induced by treatment with atezolizumab is reported.

## Case presentation

A 56-year-old Japanese man was admitted to our hospital because of high fever and consciousness disorder. He developed drowsiness and did not respond well to simple questions. He had been diagnosed with lung adenocarcinoma of the right lower lobe with upper right hilar lymph node metastasis (cT2N2M0). He was a taxi driver and had smoked until he was 55 years old (1 pack per day × 35 years). He had no other medical history or family history. He underwent right lobectomy 10 months prior to admission and had been treated with three courses of chemotherapy with a combination regimen of cisplatin plus vinorelbine, although positron emission tomography showed disease progression with multiple new metastatic lung lesions. Subsequently, he received three cycles of chemotherapy with a combination regimen of carboplatin plus nab-paclitaxel. He had received his first treatment with atezolizumab 17 days earlier for metastatic lung adenocarcinoma. His fever occurred about 1 week prior to admission. Neurological examination showed a consciousness disturbance (Glasgow Coma Scale E3V3M6) and motor aphasia. He did not show signs of pyramidal tract involvement, involuntary movement, ataxia, sensory disturbance, or autonomic disturbance. No other abnormal findings, including nuchal rigidity, were found.

**Fig. 1:**
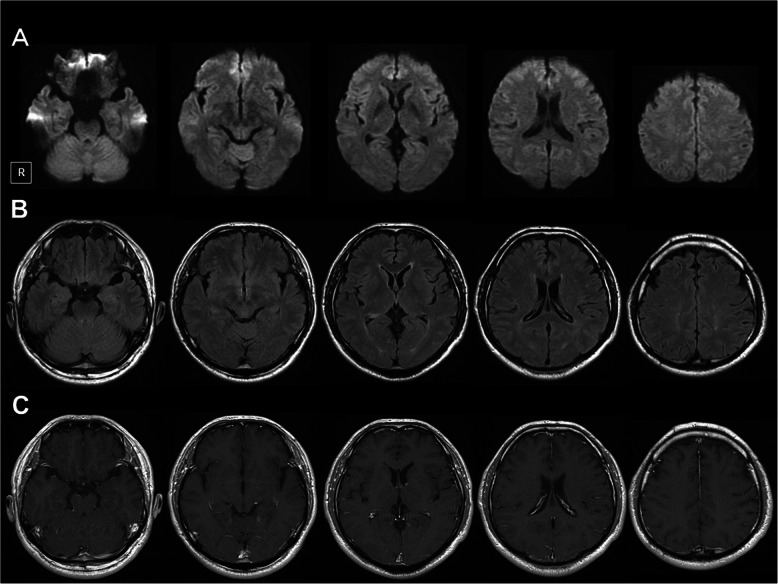
Brain magnetic resonance imaging on admission. There are no abnormal findings on (**a**) diffusion-weighted images, (**b**) fluid-attenuated inversion recovery, and (**c**) T1-weighted images with gadolinium enhancement

**Fig. 2:**
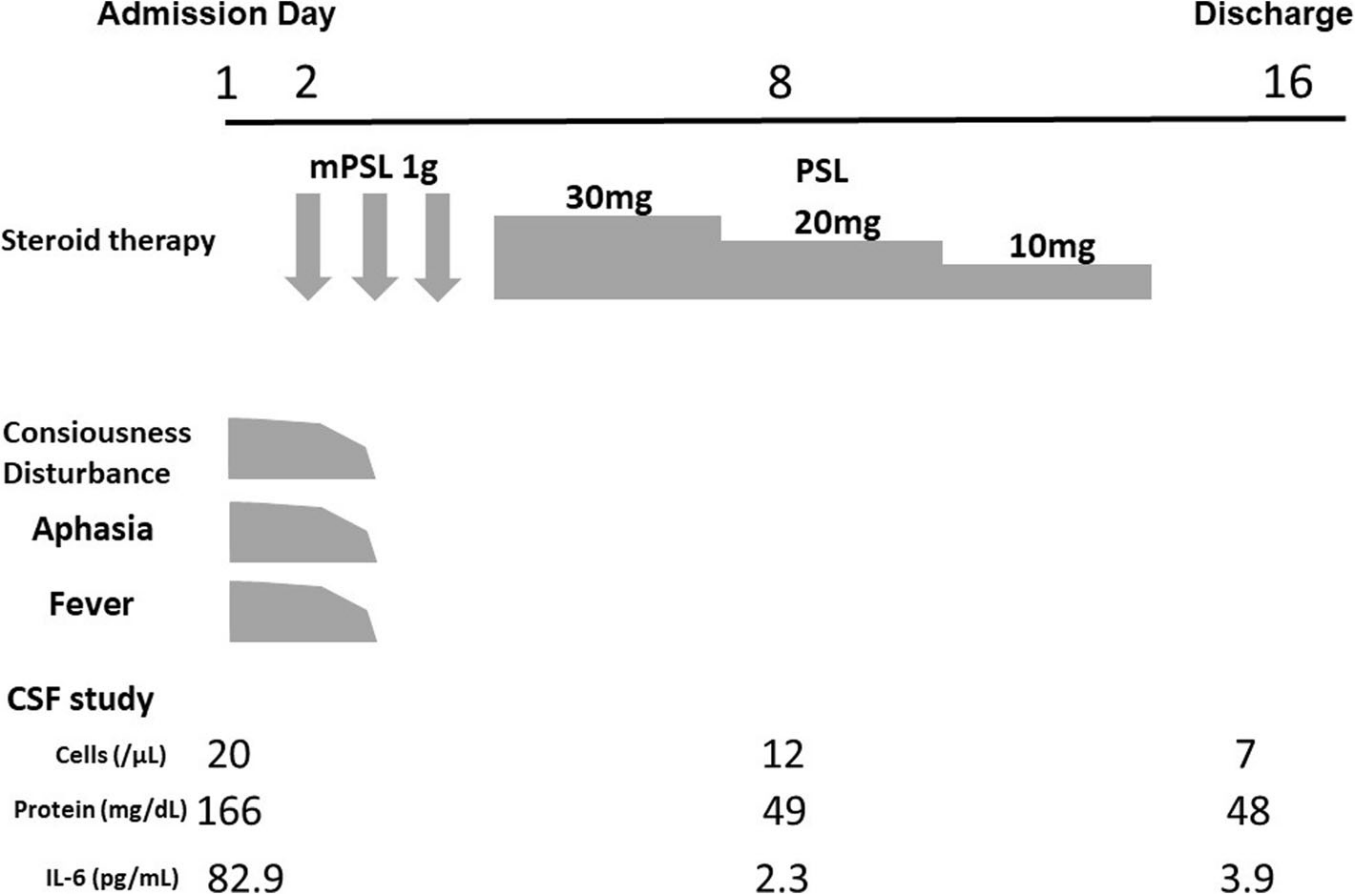
Clinical course of the present case. *CSF* cerebrospinal fluid; *IL-6* interleukin 6; *mPSL* methylprednisolone; *PSL* prednisolone

Magnetic resonance imaging with gadolinium contrast of the brain on admission showed no abnormalities (Fig. 1). A cerebrospinal fluid (CSF) study demonstrated a cell count of 20/μl, protein of 166 mg/dl, and glucose of 73 mg/dl. The level of interleukin 6 (IL-6) in CSF was increased to 82.9 pg/ml (normal level < 8.7 pg/ml [3]). The CSF was negative for bacterial cultures and polymerase chain reaction for herpes simplex viruses 1 and 2 and cytomegalovirus. Serum antibody tests for paraneoplastic neurological syndrome including anti-Hu were negative. Thus, metastatic brain tumor, bacterial meningitis, herpes simplex encephalitis, and paraneoplastic neurological syndrome were ruled out. Our patient was diagnosed with encephalitis induced by atezolizumab, and steroid pulse therapy with 1000 mg of methylprednisolone for 3 days was started on the second hospital day. His symptoms including high fever, consciousness disturbance, and motor aphasia improved immediately the next day. After the steroid pulse therapy, oral administration of prednisolone 30 mg (0.5 mg/kg) was started and tapered. The CSF findings, except for mild pleocytosis (12/μl), were normalized on day 8. The value of IL-6 was decreased to 2.3 pg/ml. Oral administration of prednisolone ended on day 13. A subsequent CSF study on day 14 showed an almost normalized cell count (7/μl) and a normalized value of IL-6 (3.9 pg/ml) (Fig. 2). He was discharged on day 16 without neurological sequelae. After discharge, he was treated with combination chemotherapy with a regimen of docetaxel plus ramucirumab for lung adenocarcinoma without relapses of encephalitis.

## Discussion

A case of encephalitis that occurred after treatment with atezolizumab was presented. Prompt diagnosis and initiation of steroid pulse therapy were successful. Long-term oral administration of prednisolone was not required. The CSF level of IL-6 reflected the severity of the encephalitis well.

Encephalitis associated with atezolizumab has rarely been reported as an irAE; to the best of our knowledge, only three cases have been reported [4–6]. Encephalitis was not reported as an irAE for atezolizumab in Phases 1 and 2 of the POPLAR trial (atezolizumab vs. docetaxel for patients with previously treated non-small cell lung cancer). On the other hand, in the OAK trial, a randomized, phase III study (atezolizumab vs. docetaxel in patients with previously treated non-small cell lung cancer), 5 of 609 patients (0.8%) treated with atezolizumab developed encephalitis [7]. Additionally, in the Impower 150 study, a randomized, phase III study (atezolizumab in combination with carboplatin plus paclitaxel with or without bevacizumab vs. carboplatin plus paclitaxel and bevacizumab), 1 of 373 patients (0.3%) developed encephalitis [8]. These patients developed encephalitis about 2 weeks after treatment with atezolizumab and showed fever and consciousness disorder, except for one who had a normal temperature [5]. CSF pleocytosis and elevated protein levels are common. Leptomeningeal enhancement or lesions of the brain parenchyma on brain magnetic resonance imaging were observed, except that two showed no abnormal findings, as in the present case. Although the management of encephalitis associated with atezolizumab has not been well-established, responses to steroid therapy were good, and further additional treatment was not required [6]. On the other hand, in some cases of encephalitis associated with nivolumab, a PD-1 inhibitor, additional treatment with immunoglobulin, or plasmapheresis was required [9, 10].

The precise pathophysiology of irAEs remains uncertain. Some potential mechanisms include increased T-cell activity against antigens that are present in tumors and healthy tissue, increased levels of pre-existing autoantibodies, increased levels of inflammatory cytokines, and enhanced complement-mediated inflammation due to direct binding of an antibody against cytotoxic T-lymphocyte antigen 4 (CTLA-4) with CTLA-4 expressed on normal tissue [1]. In the present case, the level of IL-6 in CSF was elevated in the acute phase and normalized after steroid therapy. To the best of our knowledge, this is the first case of encephalitis due to immune checkpoint inhibitors in which the level of IL-6 in the CSF was measured. Because IL-6 in the CSF is a representative cytokine reflecting inflammation in the central nervous system [3], excessive production of inflammatory cytokines was likely the cause for developing encephalitis in the present case. Increased autoantibodies may also be a possible mechanism, since one case report of encephalitis associated with nivolumab had N-methyl-D-aspartate receptor antibodies [9], but specific autoantibodies for developing encephalitis were not found in the present case.

In conclusion, a case of encephalitis associated with atezolizumab was presented. Prompt steroid pulse therapy led to a successful response, and the outcome was good. The CSF level of IL-6 reflected the severity of the encephalitis well. Clinicians should be aware of the possibility of encephalitis after initiation of immune checkpoint inhibitors. Because case reports of encephalitis associated with immune checkpoint inhibitors are very few, further investigation will be required to establish effective treatments for such life-threatening irAEs.

## Data Availability

All data generated or analyzed during this study are included in this published article.

## Abbreviations

CSF: Cerebrospinal fluid
CTLA-4: Cytotoxic T-lymphocyte antigen 4
IL-6: Interleukin 6
irAE: Immune-related adverse event
PD: Programmed death
PD-L: Programmed death ligand
